# Is central obesity associated with poorer health and health-related quality of life in primary school children? Cross-sectional results from the Baden-Württemberg Study

**DOI:** 10.1186/1471-2458-13-260

**Published:** 2013-03-22

**Authors:** Dorothea Kesztyüs, Tamara Wirt, Susanne Kobel, Anja Schreiber, Sarah Kettner, Jens Dreyhaupt, Reinhold Kilian, Jürgen M Steinacker

**Affiliations:** 1 Division of Sports and Rehabilitation, Department of Internal Medicine II, Ulm University Medical Center, Frauensteige 6, 89075 Ulm, Germany; 2 Institute of Epidemiology and Medical Biometry, Ulm University, Ulm, Germany; 3 Department of Psychiatry II, Ulm University, Günzburg, Germany

## Abstract

**Background:**

Childhood obesity and its consequences are a growing threat to national economies and health services. The aim of this study was to determine associations between waist-to-height ratio (WHtR) as a measure of central obesity, and health-related quality of life (HRQoL) and absenteeism of primary school children in the state of Baden-Württemberg, Germany.

**Methods:**

Cross-sectional data from 1888 first and second grade children (7.1±0.6 years) participating in the baseline measurements of the Baden-Württemberg Study were analyzed. Parents completed questionnaires including a rating of their children’s HRQoL using KINDL^*R*^ and EQ5D-Y VAS. Days of absence because of illness, and number of visits to a physician during the last year of school/kindergarten were asked, as well as the number of days parents took off work to care for their sick child. Anthropometric measurements were taken by trained staff. The Mann-Whitney-*U* test was used for statistical analysis of differences between WHtR groups. Logistic regression models were used to identify factors associated with sick days.

**Results:**

A total of 158 (8.4%) children were centrally obese (WHtR ≥0.5). These children had significantly more sick days (9.05 vs. 6.84, *p* < 0.001) and visits to a physician (3.58 vs. 2.91, *p* < 0.05), but not days of parental absence than other children. According to regression analysis, sick days were also associated with age, migration status, physical activity pattern, maternal health awareness and family education level. Parent-rated HRQoL was significantly lower in centrally obese children for the EQ5D-Y VAS (88.1 vs. 91.6, *p* < 0.001), and the KINDL^*R*^ subscales ’school’ (79.9 vs. 82.5, *p* < 0.05) and ’friends’ (75.4 vs. 78.3, *p* < 0.05), but not for the total score.

**Conclusions:**

Cross-sectional results show higher rates of absence, more visits to a physician and lower HRQoL in children with central obesity. Each missed day at school implies a hazard to academic achievement and each additional visit to a physician is related to higher health care costs. Thus, the negative impact of central obesity is already measurable in primary school children, which emphasizes the urgent need for early delivery of health promotion and targeted prevention.

## Background

Overweight and obesity and their associated health outcomes threaten economies and health-care systems all over the world [1-5]. As there is some evidence that the origin of overweight and obesity is often established in childhood [6], primary school children are increasingly the focus of research. Data from the German KiGGS study (German Health Interview and Examination Survey for Children and Adolescents) showed that for both boys and girls the proportion of overweight increased from 10% in 2–6-year olds to over 15% in 7–10-year-olds up to 17% in 14–17-year-olds [7]. According to this, the greatest increase in the development of overweight takes place at an age between 7 and 10 years, when children in Germany are attending primary school.

Economic consequences of the childhood obesity epidemic, particularly medical costs, have been addressed by several authors [1-5]. The direct costs of childhood obesity, including annual drug prescription, emergency room, and outpatient costs, in the US amount to ¡DOLLAR/¿14.1 billion annually [2], plus inpatient costs of ¡DOLLAR/¿237.6 million (all quotations in 2005 dollars) [3]. These sums correlate with the findings of an Israeli work group, providing objective, clinical evidence that obesity in children is associated with increased health care use [4]. Results from other studies in Germany and the Netherlands confirm this association [8,9].

Only a few authors have addressed health-related absence from school in overweight and obese children, with heterogeneous conclusions. Wijga et al. found obesity to be significantly associated with more school absenteeism [9], while Rappaport et al. only detected strong associations among extremely obese children [10]. Baxter et al. saw no significant relationship between children’s absenteeism and body mass index (BMI) [11], but Li et al. showed that increased body weight was independently associated with severe school absenteeism in children [12]. Data on parental work absenteeism because of illnesses in their overweight or obese children were only found in one study, dealing with another age group (9 to < 12 years) [13].

Sick days and illness have an impact on health-related quality of life (HRQoL). Obesity in adults is associated with impaired HRQoL [14]. There is also some evidence that HRQoL in overweight and obese children is lower than in their normal weight peers, but differences are not always significant and seem to depend on the method of rating HRQoL [15-17]. Parent-rated HRQoL is often poorer than self-rated HRQoL in overweight and obese children [17]. The KINDL^*R*^ (Revidierter KINDer Lebensqualitätsfragebogen) questionnaire is currently the most broadly used HRQoL instrument for children and adolescents in Germany, and has been translated into many languages (http://www.kindl.org) [18]. The KINDL^*R*^ includes indicators for physical, psychological, family, social and school well-being, and self-esteem [18]. Arif et al. found that overweight children have lower proxy-rated HRQoL in KINDL^*R*^ total score, and scores for subscales ’self-esteem’ and ’friends’ [19]. In their review, Tsiros et al. stated that increasing weight status had a negative impact on overall pediatric HRQoL, in which physical and social functioning appeared to be most affected [17]. Only a little information about HRQoL in children with central obesity is available, but, like in obesity classified by BMI [15-17], HRQoL can be expected to be lower.

The aims of this cross-sectional study were to examine the basic measurements of the Baden-Württemberg Study carried out in fall 2010 with regard to visits to a physician, absenteeism, and HRQoL of centrally obese primary school children. The focus was on central obesity, not only because of some practical advantages of this measure [20], but mainly because waist-to-height ratio (WHtR) and waist circumference (WC), seem to be better predictors of weight-related health risks in children (5–16 years) than obesity as determined by BMI [21,22]. Furthermore, central body fat is associated with various cardiometabolic risk factors such as insulin resistance, higher blood pressure, unfavorable lipid levels, and elevated high-sensitivity C-reactive protein concentration even in childhood and adolescents [23-26]. Indeed there is some evidence that the epidemic of obesity defined by BMI is plateauing [27], but at the same time there is doubt the same applies to central obesity [28].

## Methods

### *”Komm mit in das gesunde Boot - Grundschule”* (Join the Healthy Boat - Primary School)

Based on the positive results of the URMEL-ICE study regarding a reduction in the increase in waist circumference and favorable cost-effectiveness [29,30], a health-promotion program for primary schools in the state of Baden-Württemberg (South-Western Germany) was developed at Ulm University and has been implemented from 2009. Primary school children in grades 1 to 4 are included. The program combines behavioral and environmental measures, with the aim of changing children’s behavior and focusing on the prevention of overweight and obesity among other health-related outcomes. The lecture material is integrated into the usual curriculum and does not require additional lessons. Three crucial risk factors for childhood overweight and obesity are targeted: physical activity, consumption of sweetened beverages, and use of screen media [31]. The intervention consists of 20 units of regular teaching time spread over 36 weeks in one school year including regular activity breaks, six family homework assignments to be completed by the children and their parents, and information material for parents. Teachers are trained in three courses to familiarize themselves with the material and the implementation of the intervention. To assess the initial situation and to study the impact of the teacher-driven intervention, a randomized, controlled trial started in 2010. Only schools who had not yet participated in the program were included. The study protocol was approved by the ethics committee of Ulm University in June 2009 (Application No. 126/10). The Baden-Württemberg Study is registered at the German Clinical Trials Register (DRKS), Freiburg University, Germany, under the DRKS-ID: DRKS00000494. A detailed description of this study (”Baden-Württemberg Study”) has already been published elsewhere [32].

### Participants and data

Written informed consent was obtained from parents of 1968 pupils, representing about 62% of all eligible children. Baseline measurements of height, weight and WC were taken in 1947 children prior to the start of the intervention and the subsequent parental questionnaire was available from 1714 participants. All subsequent analyses are based on a sample size of 1888 participants providing data on sex, age, weight, height, and WC.

#### Demographics

The parental level of education was assessed and assigned to the respective level according to the CASMIN classification [33]. Family education level was determined as the highest level of two parents or the level of a single parent who cared for the child. Family education was dichotomized for analysis; elementary and intermediate education levels were clustered together in one group, tertiary level in another. The child’s migration background was defined as at least one parent being born abroad or at least one parent mainly having spoken a foreign language during the child’s first years of life.

#### Health behaviors

Parents gave information about maternal smoking during pregnancy, gestational diabetes, gestational age at delivery, birth weight and breastfeeding. They were asked to rate the sporting activity of their children, how much time a day they spent playing outside, on how many days a week they were physically active on a moderate to vigorous level for at least 60 minutes a day, the frequency of consumption of soft drinks at school and at home, and whether they had breakfast before school. Parents gave information about their own health behavior such as smoking, and were asked to rate their health awareness.

#### Anthropometric measurements

Anthropometric measurements of the children were taken by staff trained to ISAK-standards [34] in a consistent manner [32]. The children’s BMI was calculated as weight divided by height squared (kg/m^2^), and percentiles were allocated according to German reference data with 90th and 97th age- and sex-specific percentiles used as cut-off points to define overweight and obesity, and the 10th percentile was used to define underweight [35]. WHtR was calculated as the ratio of WC to height in centimeters; participants with WHtR ≥ 0.5 were categorized as centrally obese [21].

Parental BMI was calculated from self-reported weight and height data in the questionnaires, and categorized as overweight (BMI > 25.0) and obese (BMI > 30.0), according to the international classification of the World Health Organization (WHO) [36]. Parental WHtR was calculated as the ratio of self-reported WC to height.

#### Health and health-related quality of life

Parents were asked to recall the number of days their children were absent from school or kindergarten because of illness, and the number of visits to a physician during the past year. Working parents indicated the number of days they had to take off work to care for their sick child.

To assess the HRQoL of the children, parents were asked to complete the KINDL^*R*^ proxy version [37] and a visual analogue scale (VAS) taken from the EQ5D-Y Proxy Version [38], both scales ranging from 0–100. The KINDL^*R*^ questionnaire is a frequently used, validated instrument for the measurement of HRQoL in children [39]. The discriminative ability of the EQ5D-Y VAS in terms of obesity has already been successfully tested by Sach et al. [40].

### Missing data

Common to observational studies is the problem of missing data. To examine baseline differences between records with and without missing outcome variables (WHtR, absenteeism, visits to the physician, KINDL^*R*^ total score, VAS) the Mann-Whitney-*U* test for continuous data or the Fisher exact test for categorical data were used. The same applied for differences between records with and without missing independent variables.

### Statistical analysis

Baseline characteristics for anthropometric measurements were available from 1888 participants including information on WHtR. Pregnancy and birth characteristics, parental characteristics, children’s lifestyle and health characteristics were available from 1714 parental questionnaires. Correlations between parental and children’s weight measurements were calculated using Pearson’s r-statistic. Sample size in the analyses varied because of incomplete data. All analyses were carried out using the statistical software packages IBM SPSS release 19.0.0.2 for Windows (SPSS Inc, Chicago, IL, USA) and R release 2.13.0 for Windows (http://cran.r-project.org).

#### Differences between WHtR groups

Differences between the WHtR groups for all variables were tested (according to scale level and distribution of the data) using Fisher’s exact test for categorical data and Mann-Whitney-*U* test for continuous data. The significance level was set at *α*< 0.05 for two-sided tests.

#### Analysis of differences in health-related quality of life

Mean values of EQ5D-Y VAS, and KINDL^*R*^ total score and subscale scores were compared between WHtR groups. Because of the marginal non-normality of the underlying distributions, the Mann-Whitney-*U* test was applied to determine differences.

#### Analysis of absenteeism

The number of children’s sick days was dichotomized by the median value of 5 days into a group with a lower amount of sick days (≤ 5) and a group with a higher amount of sick days (> 5), because of the non-classifiable distribution of this variable. Stepwise backward elimination was used in the logistic regression to identify factors associated with sick days, and to compute adjusted odds ratios and 95% confidence intervals (95% CI).

## Results

### Baseline characteristics

**Figure 1:**
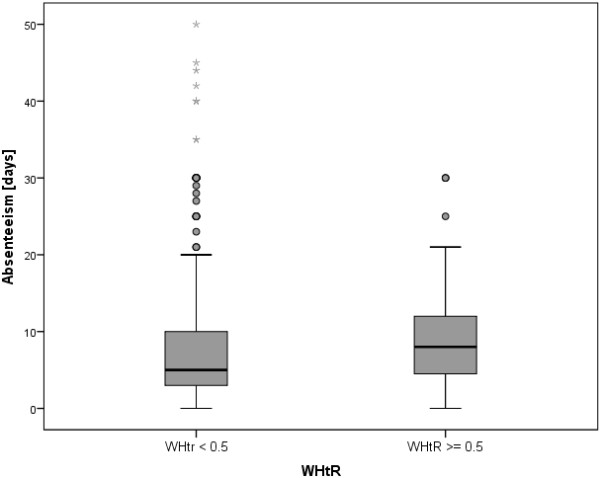
**Distribution of absenteeism in the two WHtR categories.** Box and whisker plots of the distribution of absence days in the centrally obese (WHtR ≥ 0.5) and the other (WHtR < 0.5) group.

The mean age of the included first and second grade children was 7.1 ± 0.6 years, ranging from 5.4–9.8 years, and 51.2% were boys. Table 1 presents the characteristics of the participants, including information about missing values. Significant differences between the WHtR groups were found for a number of variables. There were more children with a migration background in the group with high WHtR (*p* < 0.001). BMI, BMI percentile and WC were higher in the high WHtR group (*p* < 0.001), with no underweight and fewer normal weight but more overweight and obese children in the high WHtR group (*p* < 0.001). More mothers had smoked during pregnancy (*p* < 0.01) and children were less often breastfed in the high WHtR group (*p* < 0.05). Children with a high WHtR more often had a single parent (*p* < 0.05) and a lower family education level (*p* < 0.01). Parental overweight and smoking were more often present in the high WHtR group (*p* < 0.001). Maternal WHtR was higher (*p* < 0.001) in the high WHtR group, as was paternal WHtR (*p* < 0.01). Absenteeism from school (*p* < 0.001) and the number of visits to a physician (*p* < 0.05) were higher in the high WHtR group. Children in the high WHtR group were less often rated as sportingly active (*p* < 0.05), more often consumed soft drinks outside school (*p* < 0.01) and more often had no breakfast before school (*p* < 0.001). Figure 1 illustrates the differences in the distributions of absenteeism between the two WHtR groups using box and whisker plots. Maternal and paternal BMI correlated with children’s BMI (r = 0.28 and 0.25, respectively), and maternal and paternal WHtR were associated with children’s WHtR (r = 0.21 and 0.17, respectively, all *p* < 0.01).

**Table 1:** Baseline characteristics of participants by WHtR

	**Missing** **values**	**WHtR < 0.5** **n = 1730**	**WHtR ≥ 0.5** **n = 158**
Boys, n (%)	0	894 (51.7)	73 (46.2)
Age, years [m (sd)]	0	7.07 (0.63)	7.16 (0.71)
Migration, %	293	30.4	48.3***
**Anthropometric measures**			
BMI, kg/m^2^ [m (sd)]	0	15.57 (1.42)	21.00 (2.76)***
BMI, percentile [m (sd)]	0	44.85 (25.29)	94.23 (7.62)***
WC, cm [m (sd)]	0	54.37 (3.83)	68.86 (7.74)***
Underweight, %	0	8.6	0***
Normalweight, %	0	88.0	17.1***
Overweight, %	0	3.2	32.9***
Obesity, %	0	0.2	50.0***
**Pregnancy and birth characteristics**			
Week of gestation, [m (sd)]	291	39.12 (2.04)	38.99 (2.23)
Gestational diabetes, %	247	3.8	7.3
Birth weight, gr [m (sd)]	226	3328.0 (561,7)	3309.4 (558.3)
Smoking during pregnancy, %	238	9.5	19.0**
Breastfeeding, %	236	84.2	76.4*
**Parental characteristics**			
Single parent, %	262	10.0	17.4*
Tertiary family education level, %	365	33.4	16.5***
Maternal overweight/obesity, %	357	29.1	55.8***
Paternal overweight/obesity, %	457	59.5	78.6***
Maternal WHtR, [m (sd)]	793	0.50 (0.07)	0.54 (0.09)***
Paternal WHtR, [m (sd)]	876	0.54 (0.07)	0.57 (0.10)*
Smoking (mother), %	281	19.4	37.5***
Smoking (father), %	349	28.2	51.8***
Maternal health awareness, %	282	58.9	55.9
Paternal health awareness, %	388	44.8	45.4
**Lifestyle and health characteristics**			
Absenteeism at kindergarten/school			
because of illness last year, [m (sd)]	335	6.84 (6.70)	9.05 (6.70)***
Mothers absenteeism at work because			
of child’s illness last year, [m (sd)]	823	2.53 (4.07)	3.16 (6.91)
Fathers absenteeism at work because			
of child’s illness last year, [m (sd)]	1053	0.56 (3.72)	0.92 (2.33)
Number of visits to a physician			
last year, [m (sd)]	348	2.91 (2.88)	3.58 (3.82)*
Sporting activity, %	289	88.4	81.1*
Playing outside > 60 min/day, %	293	69.1	63.9
≥ 4 times a week active for			
≥ 60 min/day, %	316	27.1	27.8
Soft drinks consumption > 1 time/week			
at school, %	273	7.4	10.7
outside of school, %	272	24.0	35.6**
No breakfast before school, %	235	11.8	25.2***

m (sd), mean value (standard deviation).

*** p < 0.001, ** p < 0.01, * p < 0.05.

### Missing data

Participants with missing data in the outcome variables were significantly more likely to have a migration background. Their working mothers stayed at home more often to care for their sick child, they were less often breastfed, had lower family education level, their mothers were more often overweight and smoked, they were less often categorized as sportingly active, they consumed soft drinks more often at school and at home and had breakfast less often before school.

Participants with missing data in the independent variables differed significantly in the KINDL^*R*^ total score, had more days of absence from school, their working mothers stayed at home more often to care for their sick child, they had more visits to a physician, they more often had a migration background, and their mothers were more likely to have smoked during pregnancy, they were less likely to have been breastfed, they were more likely to have a single parent and a lower family education level, parents smoked more often, they were less often sportingly active, and they consumed soft drinks at school more often.

For both groups, i.e., those with missing data in the outcome variables and those with missing data in the independent variables, there was a lower proportion of normal weight participants and higher proportion of underweight, overweight, obese and centrally obese participants.

### Health-related quality of life

**Figure 2:**
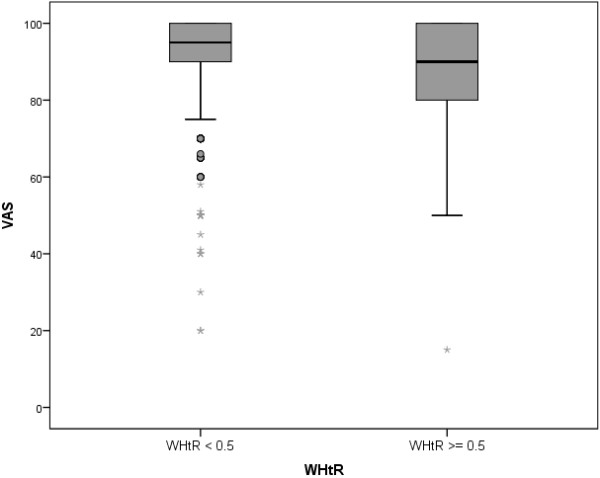
**Distribution of EQ5D-Y VAS in the two WHtR categories.** Box and whisker plots of the distribution of parent-rated HRQoL (EQ5D-Y VAS) in the centrally obese (WHtR ≥ 0.5) and the other (WHtR < 0.5) group.

HRQoL measured by EQ5D-Y VAS was significantly lower in children with higher WHtR (*p* = 0.010). These differences were also found in the KINDL^*R*^ subscales ’school’ (*p* = 0.038) and ’friends’ (*p* = 0.029). However, the KINDL^*R*^ total score did not show lower values in children with central obesity. Table 2 shows the mean values and standard deviations of the various measures. The differences in the underlying distributions of EQ5D-Y VAS ratings between the two WHtR groups are shown in the box and whisker plots of Figure 2.

**Table 2:** Children’s health-related quality of life by WHtR

	**Missing** **values**	**WHtR < 0.5** **n = 1730** **m (sd)**	**WHtR ≥ 0.5** **n = 158** **m (sd)**
EQ5D-Y VAS	261	91.63 (9.71)	88.12 (13.24)***
KINDL^*R*^ physical well-being	276	83.72 (13.43)	81.49 (14.10)
KINDL^*R*^ psychological well-being	265	84.88 (11.10)	82.71 (13.16)
KINDL^*R*^ self-esteem	226	74.01 (13.23)	74.69 (13.27)
KINDL^*R*^ family	242	80.33 (12.27)	79.06 (15.64)
KINDL^*R*^ friends	264	78.29 (12.05)	75.38 (13.44)*
KINDL^*R*^ school	223	82.47 (14.35)	79.93 (14.12)*
KINDL^*R*^ total score	351	80.72 (8.45)	79.47 (9.04)

m (sd), mean value (standard deviation).

*** *p* < 0.001, ** *p* < 0.01, * *p* < 0.05.

### Odds ratios from stepwise logistic regression analysis of higher level of child absenteeism

All variables included in Table 1, except HRQoL because of its mutual relationship with the outcome variable, were tested for significant association with child absenteeism in a logistic regression model using stepwise backward elimination. The final model consisted of age (years), WHtR (≥ 0.5), physical activity (≥ 60 for at least 4 days a week), migration background, tertiary family education level and high level of maternal health awareness. Parental weight status and WHtR were excluded to avoid collinearity with children’s weight status and WHtR, and allow use of parental health awareness as a more direct measure of parental health behavior. Unadjusted and adjusted odds ratios and confidence intervals are reported in Table 3. Age (*p* < 0.001), physical activity (*p* = 0.001), tertiary family education level (*p* = 0.001), and high level of maternal health awareness (*p* = 0.049) were associated with a lower level of child absenteeism. In addition, WHtR (*p* = 0.010), and migration background (*p* = 0.001) were associated with a higher level of child absenteeism.

**Table 3:** Odds ratios for a higher level of child absenteeism

**n = 1331**	**crude OR**	**95% CI**	**adjusted OR**^***a***^	**95% CI**
Age [years]	0.56	[0.48; 0.67]	0.52	[0.43; 0.63]
WHtR ≥ 0.5	2.15	[1.44; 3.23]	1.82	[1.16; 2.85]
≥ 60 min/day physical activity ≥ 4 days a week	0.67	[0.53; 0.85]	0.66	[0.51; 0.85]
Migration background	1.44	[1.14; 1.81]	1.55	[1.20; 1.99]
Tertiary family education level	0.65	[0.52; 0.82]	0.68	[0.54; 0.87]
High level of maternal health awareness	0.82	[0.67; 1.01]	0.80	[0.63; 0.99]

OR (odds ratio), CI (confidence interval).

^a^Adjusted for the listed variables in this table as a result of stepwise backward elimination in a logistic regression model.

## Discussion

### Critical interpretation and meaning

Children with central obesity in this cross-sectional study showed higher rates of school absenteeism, more visits to a physician, and lower HRQoL. Central obesity and migration status increased the odds of children having more than 5 days of sickness absence to 182%, and 155%, respectively. On the other hand, regular physical activity (≥ 60 min/day for at least 4 days a week) reduced the odds of a higher level of absence by 52%. Further factors reducing the odds of a high level of sickness absence were age (92% lower odds per year), maternal health awareness (25% lower odds) and family education level (47% lower odds). Regarding a higher level of sick days, parents of centrally obese children recalled significantly more visits to a physician than parents of children without central obesity. In addition, children with central obesity showed significantly lower scores for HRQoL, EQ5D-Y VAS as well as KINDL^*R*^ subscales ’friends’ and ’school’.

With regard to WHtR, no German reference data were available for the age group presented here. However, the overweight and obesity rates found in this study (5.7% and 4.4%, respectively) for children in Baden-Württemberg entering elementary school differed only marginally from the data of the reference groups collected from 1985 to 1999 [35]. Nonetheless, it is to be feared that the increase in BMI and WHtR in first and second graders has just started, as the data of the KiGGS study implied [7]. Because of the close relationship between WHtR and health risks, mainly cardiometabolic risks [22], this trend needs to be stopped. According to data published by the WHO, cardiovascular diseases are the leading cause of death in the world and diabetes mellitus is among the top ten causes in middle and high income countries [41]. In the present study, signs of morbidity, such as higher rates of absenteeism, more visits to a physician, and lower HRQoL could already be measured in primary school children.

More frequent visits to a physician imply higher health care expenses for centrally obese children. This is consistent with findings from Germany and the Netherlands, which found that obese children had significantly higher physician costs and a higher probability of being high utilizers of health care services [8]. Furthermore, Wijga et al. reported that childhood obesity was significantly associated with more visits to the general practitioner [9].

The present results indicated that parent-rated child HRQoL in the EQ5D-Y VAS was at a high level for both, centrally obese children and non-obese children. The rating for the KINDL^*R*^ total score, however, tended to be slightly higher than in the age-related reference group in the above-mentioned KiGGS study (80.6 vs. 79.0) [42]. Lower HRQoL found in the subscales ’school’ and ’friends’ may be related to more visible signs of central obesity such as a large belly, and children can be very cruel in pointing out what makes other children different from themselves [43]. Lower values in the KINDL^*R*^ subscale ’friends’ for overweight children have already been found in a previous study [19]. Another reason for lower values in the subscales ’school’ and ’friends’ may be the stigmatization of overweight and obese children, with negative social and emotional consequences [44]. Additionally, central obesity may slow down children or discourage them from taking part in active play during break time and physical education lessons [45].

The strongest factor associated with a higher level of sickness absence besides age was central obesity, according to WHtR beyond the cut-off point. Children’s physical activity level was another modifiable factor identified in this study. According to Kim and Lee, leisure time physical activity is associated with abdominal obesity in youth [46], so it is understandable that both variables were significantly associated with higher levels of sickness absence. Furthermore, many authors have already addressed physical activity as an important determinant of physical and psychological health, suggesting a dose-response relationship in observational studies with more physical activity resulting in greater health benefits [47,48]. Maternal health awareness, a further modifiable factor identified in the present research, may directly influence a child’s health, as the mother usually is the most important care giver in maintaining the well-being of the child for whom she cares. Migration status and family education level are unchangeable components, and both are also known risk factors for childhood overweight and obesity [49]. Migration and lower levels of education may lead to less concern about health behaviors, resulting in a greater chance of more sick days. Age is constantly increasing and as children grow older, their immune system is believed to grow stronger so that their parents may have fewer concerns about their health.

BMI and age- and sex-adjusted BMI percentiles, in contrast to WHtR, showed no significant relationship with a higher level of sickness absence. This finding correlates with a study of more than 20 000 workers in Belgium where central abdominal fatness, but not BMI, was found to be an independent predictor of sick leave [50]. Rappaport et al. found that race and poverty appeared to affect absences to a greater extent than overweight and obesity as classified by BMI in Philadelphian school children, grades 1–12 [10]. Baxter et al. did not find significant relationships between absenteeism and age- and sex-specific BMI percentiles, and socio economic status, but found a significant inverse relationship between absenteeism and academic achievement in fourth grade children [11]. Obesity was significantly associated with a lower general health score, more visits to a physician, more school absenteeism and more health-related limitations in a Dutch study with 8-year old children [9]. These heterogeneous findings underscore the need for further investigation of the relationship between anthropometric measures and absenteeism.

Central obesity seems to encapsulate the health risks of obesity [51], otherwise one would not expect to find the reported associations at an early stage in such a young population. Children starting school are subjected to a change of lifestyle with increasing sedentary behavior and decreasing physical activity levels [52], because of the essential demands of the current character of schooling. Higher levels of sickness absence are substantially consistent with lower levels of HRQoL and do not need to be explained any further. Thus, by promoting physical activity in school and reducing time spent sedentary, central obesity may be reduced as well as sick days, and consequently HRQoL may be increased.

### Strengths and limitations

One strength of the study presented in this article is the inclusion of a complete federal state of Germany. Spread over the entire territory of the state of Baden-Württemberg, a school-based health-promotion program, developed and disseminated by Ulm University Medical Center, was evaluated in fall 2010 and 2011. Teachers of 157 classes with 1968 pupils at 84 schools decided to take part in this study. Data from all parts of the state could be collected to provide a descriptive picture of the situation of primary school children today. Another strength is the quality of the target group’s anthropometric measurements according to ISAK-standards. A further strength is the high return rate of parental questionnaires (87%). In addition, the application of two independent instruments for the assessment of children’s HRQoL enabled differentiated valuation. The EQ5D-Y VAS offers an overall measure of global health whereas the KINDL^*R*^ results in a profile of HRQoL, allowing the examination of aspects such as physical and emotional well-being, self-esteem, family, friends and school.

There are restrictions in an observational study compared with a clinical study, and the present research shows some limitations concerning missing data and selection bias. Missing data in various variables diminished the number of subjects in the logistic regression from 1888 to 1331 (70.5%). Missing data may have led to a form of selection bias but, in the best case, would only lessen the precision of the study [53], and according to the differences observed in the analyses of missing data in this study, would have underestimated the scale and significance of the results. As the analysis of missing data implied, participants with missing data predominantly showed an unfavorable profile in the critical variables: higher proportion of migrants, lower family education level, more absence of working mothers, children less often sportingly active and a higher proportion of mothers who smoked. If those participants could have been included in the analysis, this probably would have meant stronger evidence for the present results.

Another limitation concerns the quality of the parent-reported data. Parents may retrospectively have forgotten the actual number of days their child could not go to school or kindergarten during the past year because of illness, and the correct number of visits to a physician, especially if they had more than one child. Parents self-reported weight, height and WC may also have been less exact than the according-to-protocol anthropometric measurements of their children. Physical activity patterns of the children asked in the parental questionnaire were only vague approximations of real activity levels measured by accelerometers as the objective method of choice. Furthermore, pediatric HRQoL based on a proxy perspective is controversial, but analysis of the KINDL^*R*^ revealed an acceptable accordance between parental and child reports [37]. Whenever possible, both sources of information should be used. Since more than half of the children in this study were aged 7 years and younger, and had just started school, the researchers abstained data from self-reported HRQoL in the baseline measurements in 2010. All teachers opted in voluntarily and were likely to be engaged in health-promotion from the outset, thus it can be assumed that the study population differed from the basic population. The teachers would particularly influence longitudinal data and effects, but were likely to have less influence on this cross-sectional baseline analysis.

## Conclusions

Considering all the consequences of the overweight and obesity epidemic, and the fact that the development of overweight starts in childhood, the early introduction of preventive measures for all subjects at risk, which means all children exposed to a modern lifestyle, is called for. The impact of central obesity on health and HRQoL can already be seen in first and second grade school children before or at the very start of the upward trend in weight gain [7]. Also higher rates of school absenteeism, like those found in the present study, are associated with lower academic achievement [11]. Moreover, childhood obesity is generally associated with lower parental education level and socio-economic status [6-8], thus without early school-based measures it may become a vicious cycle of obesity and low education attainment [54]. Primary school children, especially prepubertal children, may still be in a more impressionable phase with a good chance of establishing a healthy lifestyle, for example because parents have a greater amount of control regarding food choices inside and outside the home [55]. Because it would involve much effort and cost for governmental authorities or health authorities to directly provide health information for each family, a cost-effective solution to teach children sustainable healthy living is to include health education into the regular curriculum in schools, especially in primary schools and to address parents as well. Teachers are key players and need to receive a science-based education emphasizing health promotion and health awareness at university to enable them to embed health education in the everyday routine of any type of school, thus implementing the request of the EU for “health in all policies” [56]. More research, preferably from representative unbiased samples, is needed to examine the association between school absenteeism, HRQoL and central obesity.

## Competing Interests

The authors declare that they have no competing interests.

## Authors’ contributions

DK, TW, SKO, AS, SKE, and other members of the research group planned and organized the Baden-Württemberg study, and were involved in carrying out the measurements in fall 2010. DK performed the statistical analyses, and JD provided expert advice. JMS is the director of the program ”Komm mit in das gesunde Boot - Grundschule” and principal investigator of the Baden-Württemberg Study. DK drafted the manuscript. TW, SKO, AS, SKE, RK, and JMS revised the manuscript drafts. All authors have read and approved the final version of the manuscript.

## Authors’ information

**Members of the “Komm mit in das gesunde Boot - Grundschule” - Research Group** Susanne Brandstetter, Clemens Drenowatz, Jens Dreyhaupt, Nanette Fischbach, Eva Maria Friedemann, Verena Hundsdörfer, Melina Klepsch, Dorothea Kesztyüs, Sarah Kettner, Benjamin Koch, Susanne Kobel, Ileana Limberger, Dmytro Prokopchuk, Anja Schreiber, Sabrina Sufeida, Olivia Wartha, Martina Wiedom, Tamara Wirt, Rainer Muche, Tina Seufert, Jürgen Michael Steinacker.

## Pre-publication history

The pre-publication history for this paper can be accessed here:

http://www.biomedcentral.com/1471-2458/13/260/prepub
